# Vertical Light Sheet Enhanced Side-View Imaging for AFM Cell Mechanics Studies

**DOI:** 10.1038/s41598-018-19791-3

**Published:** 2018-01-24

**Authors:** Kellie Beicker, E. Timothy O’Brien, Michael R. Falvo, Richard Superfine

**Affiliations:** Department of Physics and Astronomy, UNC-Chapel Hill, Chapel Hill, NC 27599 USA

## Abstract

The ability to measure dynamic structural changes within a cell under applied load is essential for developing more accurate models of cell mechanics and mechanotransduction. Atomic force microscopy is a powerful tool for evaluating cell mechanics, but the dominant applied forces and sample strains are in the vertical direction, perpendicular to the imaging plane of standard fluorescence imaging. Here we report on a combined sideways imaging and vertical light sheet illumination system integrated with AFM. Our system enables high frame rate, low background imaging of subcellular structural dynamics in the vertical plane synchronized with AFM force data. Using our system for cell compression measurements, we correlated stiffening features in the force indentation data with onset of nuclear deformation revealed in the imaging data. In adhesion studies we were able to correlate detailed features in the force data during adhesive release events with strain at the membrane and within the nucleus.

## Introduction

Cells exist in a complex physical environment where they are acted upon by, and respond to, a wide range of mechanical stimuli^1–3^. An increasing body of evidence has established links between abnormal cell mechanics and diseases states ranging from cancer^4,5^ to muscular dystrophy^6^. The mechanisms responsible for a cell’s response to external forces are especially of interest because of their downstream effects on gene expression, differentiation, and motility. Mechanotransduction, the process through which cell signaling pathways are initiated by force stimuli, starts with mechanical deformation. Understanding how a dynamic force profile alters sub-cellular structure is essential to developing a quantitative understanding of mechanotransductive response. Critical to addressing this need is the ability to collect high resolution structural data combined with application and measurement of forces. High temporal resolution with precise synchrony between the application of force and the acquisition of image data is also essential to capture structural dynamics under load.

Atomic Force Microscopy (AFM) has become prevalent in the cell biology community for its utility in probing cell mechanics^7–9^, and is often combined with fluorescent imaging for correlating structure with force data^10^. Despite the insights that wide-field plan-view epifluorescence imaging provides when combined with AFM, the method is limited because the forces are applied in the z-direction, perpendicular to the imaging plane. Thus, the most substantial cellular deformations and structural rearrangements are poorly captured in the image data. There are two common approaches for overcoming these limitations – confocal microscopy and the use of custom side-view imaging chambers using a second, laterally-oriented objective. The combination of AFM with confocal microscopy has disadvantages such as poor axial resolution, and speed; it requires seconds to collect image stacks for 3D reconstructions. Many relevant mechanical processes, such as single-cell adhesion events occur on millisecond time scales^11,12^.

A second approach is engineering a direct side-view imaging path, which leverages the full resolution and speed capabilities of the imaging system. A handful of sideways imaging systems with force measurement capability have been used to measure viscoelastic properties during cell stretching^13^, cell compression^8,14^, and cytoskeletal rearrangement^8^. However these systems have one or more the following disadvantages: loss of force sensitivity due to image based cantilever deflection measurement^13^, do not accommodate fluorescence imaging^13,14^ or are limited in ease of use and flexibility due to complex custom sample chambers^8^.

Here we describe the development of a unique vertical light-sheet illumination (VLS) and “pathway rotated imaging for sideways microscopy” (PRISM) system for use with the AFM. Our system enables simultaneous high resolution force measurements (10 s of pN) and high frame rate, high numerical aperture epifluorescence imaging of samples in the plane of dominant AFM induced stresses. Within our system, a single vertical plane of the sample is illuminated and a small mirror rotates the imaging plane of a standard epifluorescence microscope. The VLS and PRISM systems are easily integrated with a standard combined AFM inverted epifluorescence imaging system, and provide the flexibility to select any cell on a prepared sample.

We demonstrate the utility of the combined force and imaging system in studies correlating dynamic force and structural data of cells under compressive and adhesive stress. AFM derived cellular elastic modulus is typically determined by fitting the force-indentation data with the Hertz model^7^ which approximates the cell as uniform homogeneous elastic medium. In prior studies, deviations in AFM force-indentation results from Hertzian behavior have been attributed to the contribution of subcellular components such as the glycocalyx^15^, the actin cytoskeleton, the microtubule network, intermediate filaments and the nucleus^16–19^. However, these experiments either lack sufficient force sensitivity, image resolution or synchronization - both in time and space - to fully delineate these contributions. In the present work, we demonstrate our system’s ability to directly correlate distinct features in the force curve data with a transition from cytoplasmic to nuclear deformation.

For our adhesion studies, we employed our side-ways imaging system to observe intracellular strain as the AFM probe pulled away from the cell after making controlled adhesive contact. Recent studies have identified physical connections between the cytoskeleton and nucleoskeleton^20^ that mediate force transmission and can act to regulate nuclear structure and function^21^. Forces applied directly to surface integrins have been shown to produce rapid structural changes in the cytoskeleton and nucleus^19,22,23^, suggesting force transmission through direct mechanical linkage. Prior studies investigating long-range force propagation in living cells have been limited in force sensitivity, image resolution, or high-speed synchronization capabilities required to fully characterize the phenomenon. Our system enables high frame rate side-view imaging of adhesive events and nuclear strain. This allowed us to demonstrate the ability to correlate adhesive force events with cellular structural changes at the membrane and within the nucleus with 10 ms time resolution.

## Results

### Instrument design and characterization

**Figure 1:**
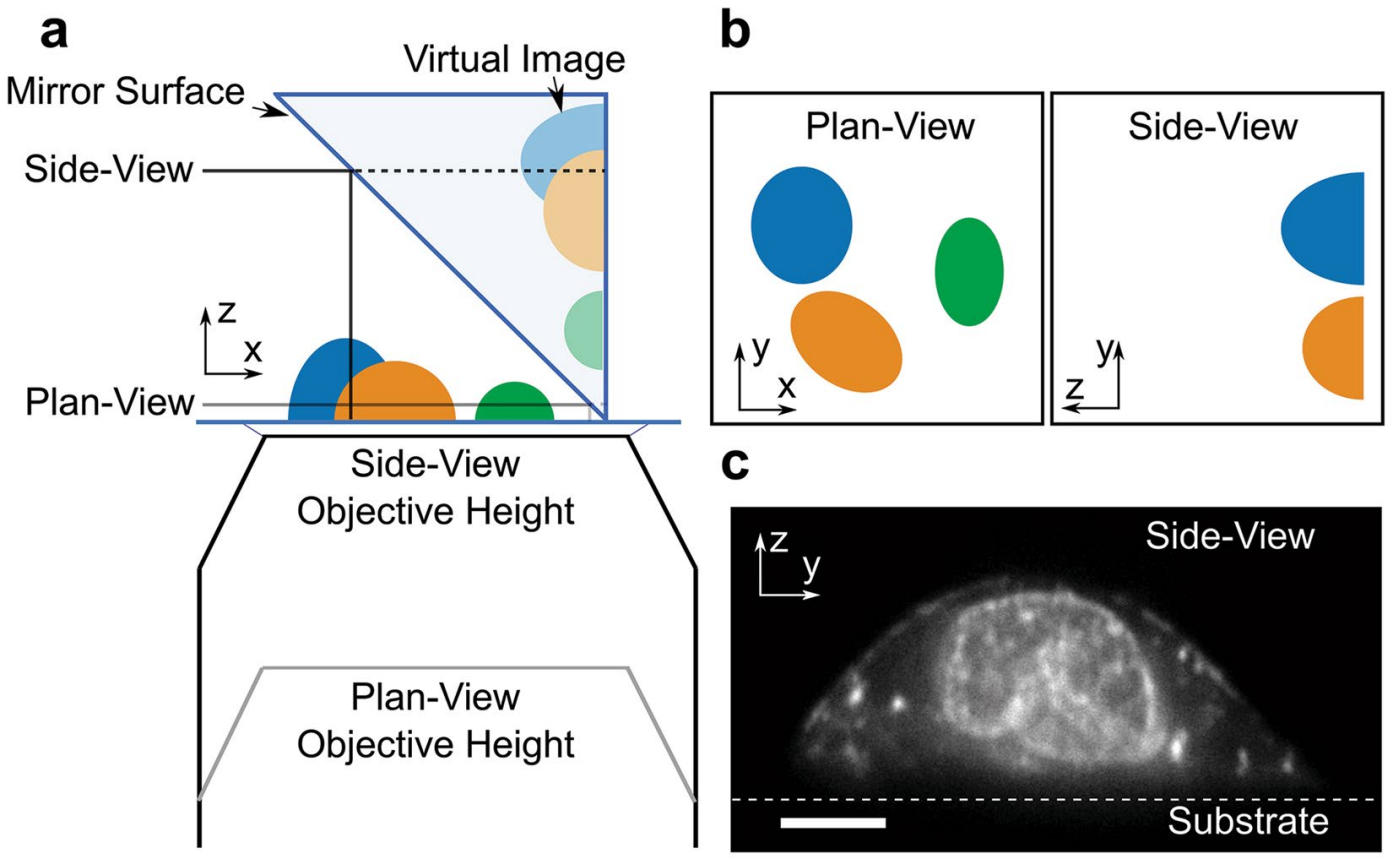
PRISM image formation. (**a**) A cartoon of the PRISM imaging system showing the mirrored surface of the 45-degree reflecting optic, objective, and cells (blue, orange, and green). Both the plan-view and reflected side-view imaging planes are indicated. (**b**) Both the plan- and side-view imaging planes as they would appear in camera view. (**c**) A side view image of an ovarian cancer cell (SKOV3) with both membrane and nucleic acids labeled (Vybrant and SYTO 83 respectively). Scale bar = 5 um.

The principle behind PRISM is straightforward: a 45° reflecting optic is freely positioned next to a fluorescently-labeled cell in the field-of-view (FOV) of an epifluorescence imaging system (See Fig. 1, Supplementary Fig. 1 and Video 1). As the objective is moved toward the sample, the object focal plane moves upwards in z, and intercepts the reflecting optic, which rotates the object plane into a vertical (y-z) orientation. As the objective is moved further toward the sample, the vertical object plane sweeps across the cell, creating vertical slices of the specimen (Fig. 1, Supplementary Video 2). The side-view image slices are displaced horizontally from the plan-view image within the FOV. The PRISM imaging system provided z-resolution in side-view images comparable to the x and y resolution achieved in standard plan-view imaging with FWHM of ~0.3 µm in z for a diffraction limited source (see Methods). We note that micro-mirror systems have been used by other researchers to measure cell behavior under flow and to create 3D reconstructions of single cells^24–30^. Similar single objective illumination and imaging systems have also been used for a variety of other cell studies^24,27,31,32^.

To reduce the contribution of out-of-focus fluorescence in the side-view image, we built an optical system that produces VLS illumination brought through the imaging objective (See Methods and Supplementary Fig. 2). The objective focuses an elliptical laser beam produced by the VLS optics to form a sheet of light with a beam waist of ~1 µm and Rayleigh length of ~5 µm, which compares favorably with the light-sheet produced by other leading systems in the field^33^. The lateral and axial position of the VLS beam waist can be controlled precisely with mirror adjustments.

**Figure 2:**
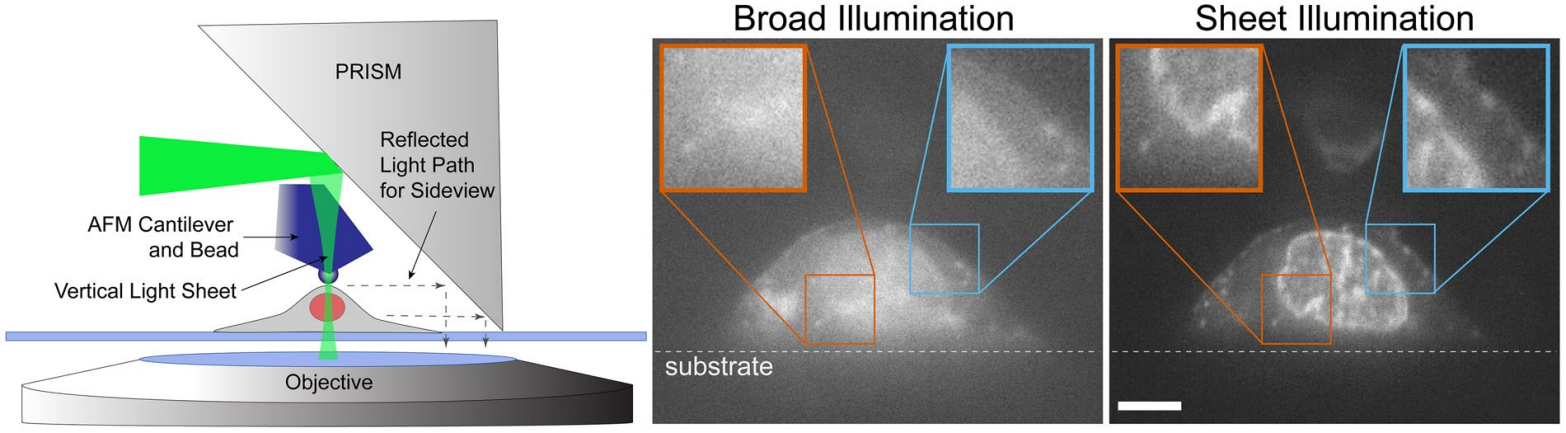
VLS enhanced PRISM imaging with AFM access. Left is a cartoon depiction of the experimental set up. The light sheet emerges from the imaging objective illuminating a vertical slice of the cell. A beaded AFM cantilever accesses the top of the cell for force measurements. A side-view image of the cell is reflected back into the objective, horizontally displaced from the plan-view image. The center and right panels show side-view images of an ovarian cancer cell labeled with membrane (Vybrant) and nucleic acid (SYTO83) dyes using broad and sheet illumination respectively. Images are taken with the same exposure time and plane of focus, and indicate a pronounced improvement in signal to noise with VLS illumination. Enlarged regions of interest show improved resolution of the nuclear boundary and cell membrane. Scale bar = 5 um.

The VLS greatly improves the signal-to-noise ratio and, thus, the ability to resolve detailed structure in VLS illuminated PRISM images as compared to broad illumination (Fig. 2 and Supplementary Fig. 3). Structural details of the nuclear envelope and cell membrane within the regions outlined by the blue and orange boxes in Fig. 2 are much better resolved. Using VLS also significantly reduced bleaching of the sample relative to broad illumination due to lower required illumination intensity as has been reported elsewhere for both Gaussian^34^ and Bessel light sheets^35,36^. Typical simultaneous AFM-PRISM/VLS results are shown in Supplementary Fig. 4 (See also Supplementary Video 3). Synchronization of PRISM imaging with AFM force data was accomplished to ≤1 ms by using the AFM controller as an external trigger for the Orca Flash 4.0 (See Supplementary Videos 4 & 5, and Methods). In practice, the frame rate for PRISM images of fluorescently labeled cell images was limited to 20 ms by the exposure time necessary for specific labels.

### Correlated Force and Sub-Nuclear Structural Dynamics

**Figure 3:**
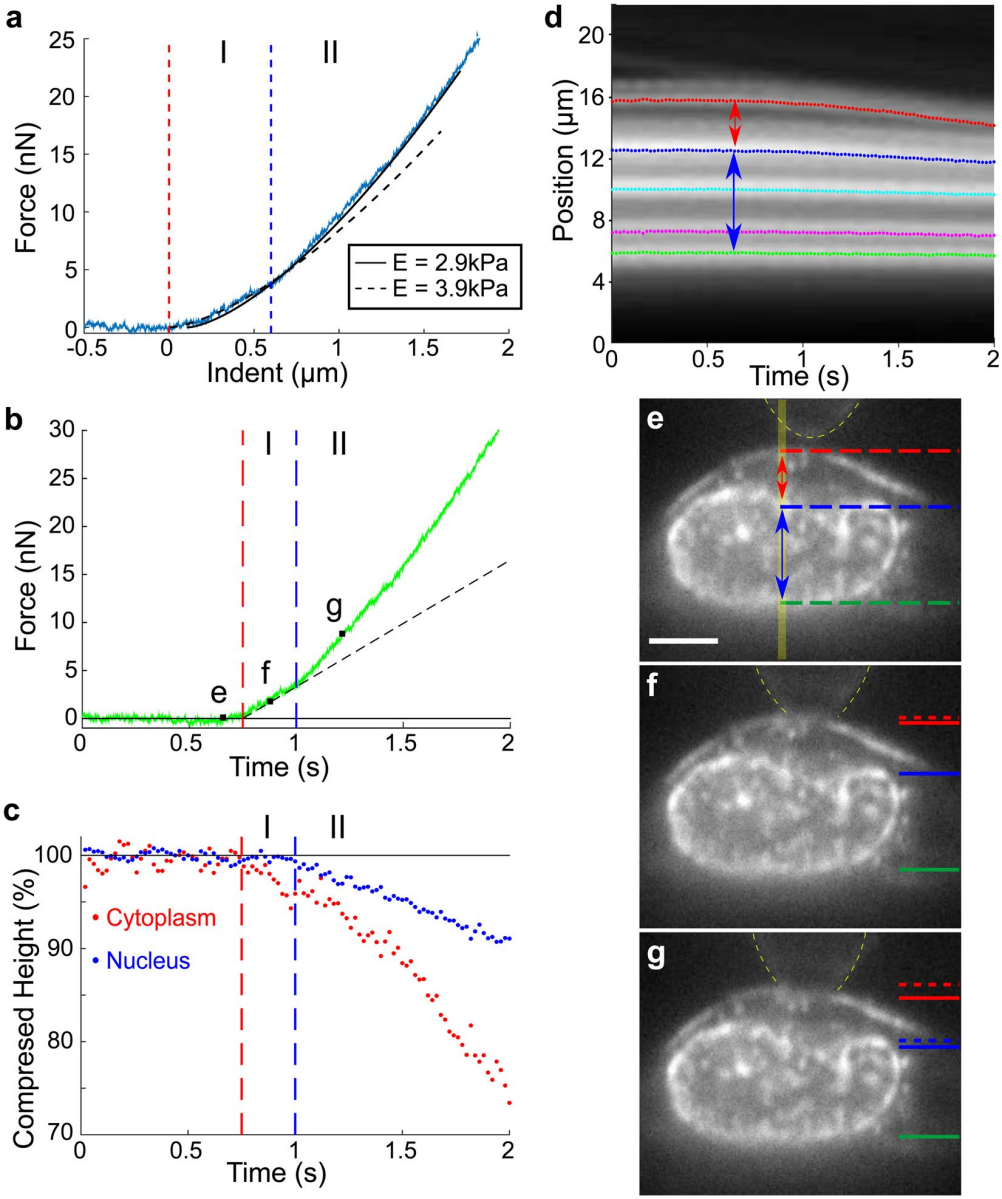
Stiffening of SKOV3 cell corresponds to onset of nuclear deformation. (**a**) AFM force vs. indentation data on SKOV3 cell fit to a two regime Hertz model, with the transition point (indicated by blue dashed line) determined through least squares fitting. (**b**) Force vs. time plot of same data as (**a**). Transition point from regime I to regime II as determined in (**a**) shows an abrupt change in slope. (**c**) Normalized compressed height vs. time of the cytoplasm region above the nucleus and the nucleus during indentation, as determined using kymograph data (**d**). The cytoplasm is defined as the region between the cell membrane (red) and the top of the nucleus (blue) indicated by red arrow in (**d**), and the nuclear region, indicated by blue arrow, is defined by the kymograph traces at the top (blue) and bottom (green) of the nucleus in (**d**). (**d**) Kymograph of the yellow region under AFM tip (indicated by vertical yellow bar in (**e**)) over the course of indentation with Gaussian tracked intensity peaks. The dotted curves correspond to the cell membrane (red), top of the nuclear region (blue), punctate labeled structures within nucleus (cyan and magenta) and the bottom of the nuclear region (green). (**e**–**g**) Sample images from a time sequence acquired simultaneously with force data, as identified on the force vs. time trace (**b**). Dashed lines on the left of the images indicate the initial position of the traced cytoplasm (red) and nucleus (blue) regions and solid lines indicate the current positions. Yellow dashed lines indicate outline of AFM mounted bead. Scale bar in (e) = 5 um.

As an initial demonstration of the combined AFM - PRISM/VLS system, we probed the mechanical response of ovarian cancer cells (SKOV3) to an applied compressive load. Simultaneous AFM force and PRISM/VLS image data were acquired for a SKOV3 cell labeled with membrane (Vybrant) and nuclear (Syto83) stains (Fig. 3). Figure 3a shows the approach and indentation portion of the AFM force curve, and representative images (e-g) from a 50 fps image sequence of the cell during deformation. We found that the force-indentation data were poorly fit by the Hertz model over the whole indentation depth, but were much better fit assuming two stiffness regimes (Fig. 3a). The stiffness regimes were determined by fitting a two-Hertz model with the transition point between regimes used as an additional fitting parameter. Regime I, the indentation region of 0 to ∼0.6 μm (corresponding to t ~0.75–1 second), yielded an elastic modulus of 2.9 kPa, and regime II, the indentation region of approximately 0.6 to 1.8 μm (corresponding to t ~1–2 seconds), yielded an elastic modulus of 3.9 kPa - a nearly 1.5-fold increase. The force vs. time data (Fig. 3b) show an abrupt change in slope at this transition point between regime I and II.

The side-view images (Fig. 3e–g) show the positions of the cell membrane, nuclear envelope and punctate structures within the cytoplasm and nucleus. The relative positions of these cellular components were obtained and compared to force data (Fig. 3b), by tracking the kymograph intensity peaks of an image slice extending below the point of contact (Fig. 3e, Supplementary Fig. 5 and Methods). The cytoplasmic region between the AFM membrane contact and the nucleus, and nuclear region are defined in Fig. 3e with the red and blue double tipped arrows respectively. Figure 3c depicts the normalized height of the cytoplasm and nuclear regions during the indentation measurement. Note that the nucleus (blue curve) shows no appreciable compression until regime II; the transition from low stiffness to higher stiffness as indicated in force data (Fig. 3a & b) is correlated to the initiation of nuclear deformation. Though the stiffening we observe has been reported in other studies^37^, our study for the first time provides preliminary structural evidence strongly implicating nuclear compression as the cause. The bottom of the nucleus shows no motion within our measurement uncertainty over the course of the experiment, which indicates no compression of the thin, unlabeled cytoplasmic region below the nucleus. Additionally, a comparison of average cytoplasm and nuclear heights before indentation, during indentation and after retraction of the AFM tip revealed no measurable changes in region height that would indicate plastic deformation (Supplementary Fig. 6).

### Nuclear Motion Linked to Membrane Adhesion

**Figure 4:**
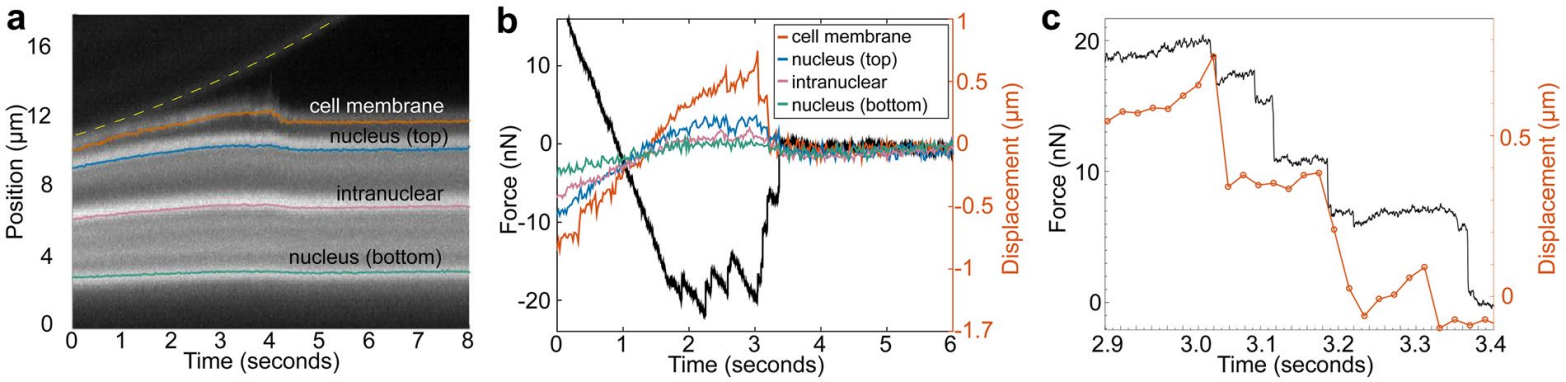
Simultaneous adhesion force and image data acquired for a fibronectin-coated AFM tip on a SKOV3 cell. (**a**) The kymograph shows motion of the AFM tip off the cell surface eventually leaving the FOV at ∼ 4 seconds (diagonal band at top highlighted with yellow dashed line), and several bright fluorescent regions of the cell - cell membrane (red), top of nucleus (green), punctate region within nucleus (cyan), and bottom of nucleus (magenta). (**b)** Time synchronized AFM force data (black - left axis) and displacements (red, green, cyan, magenta - right axis) measured from images are displayed on the same plot. (**c**) Zoom in of (**b**) with only force data (sign of force inverted for clarity) and cell membrane displacement data displayed. Some force drops are associated with membrane displacements while others are not.

Next, we examined subcellular motion due to force applied to adhesions between a fibronectin-coated bead and the cell surface. For these experiments, fibronectin on the AFM beaded tip is expected to form ligand-receptor interactions with integrins in the cell membrane, which, in turn, provide a mechanical link, via talin, to the actin cytoskeleton. The retraction portion of the force curve contains numerous inverted peaks associated with the rupture of attachments between the tip and cell (Fig. 4b). The force-rupture peaks are produced when the cantilever support is pulled away at constant velocity resulting in an increasing force on the attachments between the bead and cell until individual attachments break. The characteristic saw-tooth force pattern is caused by individual adhesions breaking in sequence.

**Figure 5:**
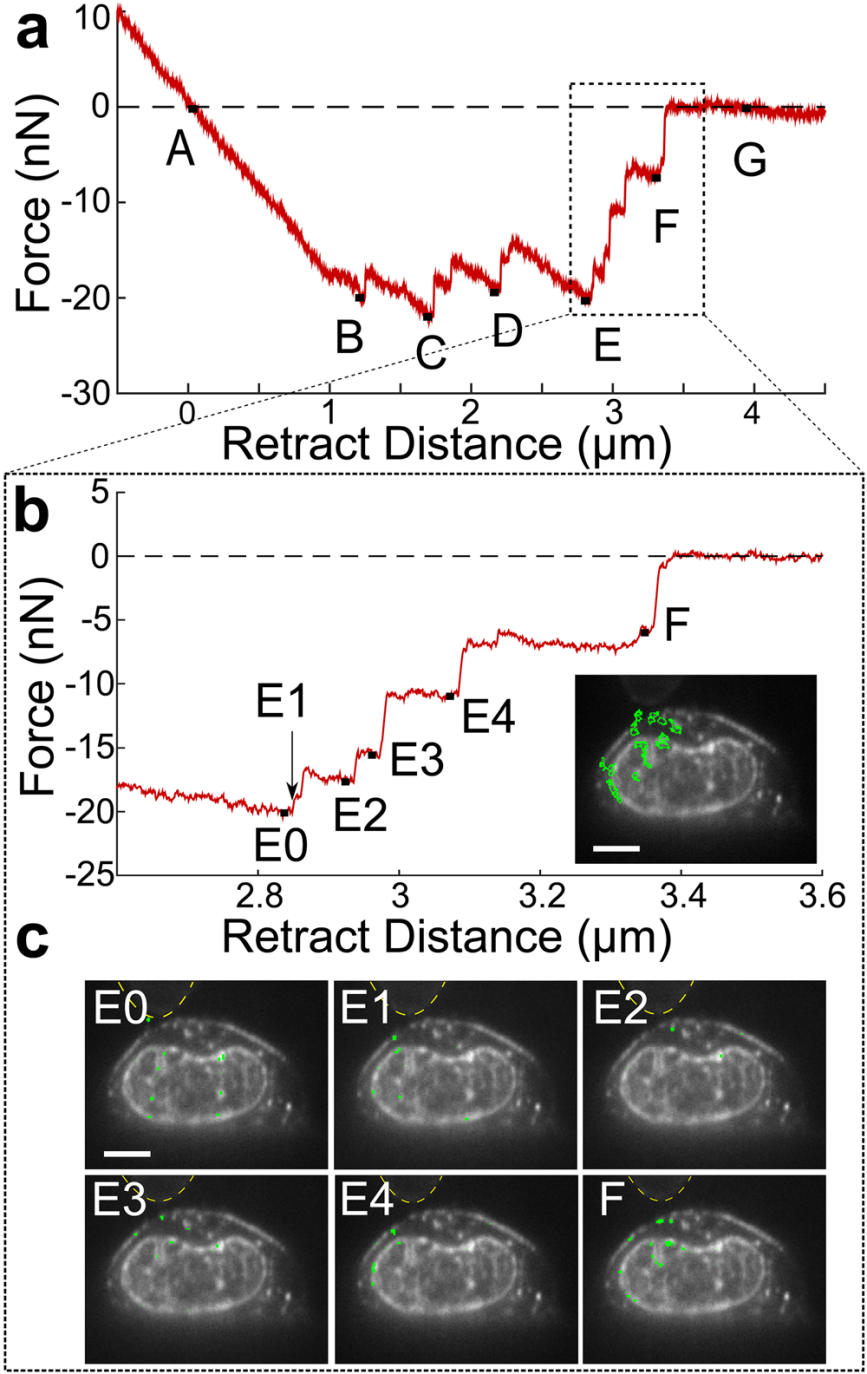
Membrane and nuclear displacements observed in response to force-rupture events between the AFM-tip and cell membrane. (**a**) Retraction portion of force-indentation curve with important points (A-G) identified. A, the point of zero force application to the cell, B-F, force-rupture peaks, and G, after bead releases from cell. (**b**) A closer examination of peaks E and F with sub-peaks of the E rupture event identified. No point is shown for E1 because this is the frame immediately following Peak E0. Inset indicates regions where displacement is measured between points E and F highlighted in green. These regions were determined through difference imaging using frames taken at E and F. (**c**) Regions of cell displacements determined through difference imaging highlighted in green for the sub-peaks indicated in (**b**). Yellow dashed lines indicate outline of AFM mounted bead. Scale bars = 5 um.

For this data set (Figs 4 and 5), we examined 10 force-rupture events, ranging in peak height from 0.1 nN up to nearly 10 nN. Each rupture event was correlated with structural changes as observed in the side-view images of the SKOV3 cell labeled with membrane (Vybrant) and nucleus (SYTO 83) dye. A kymograph for the vertical region directly under the AFM tip was acquired where structural changes were expected to be greatest. The kymograph (Fig. 4a) showed five clear bands of fluorescence representing the bottom of the AFM-mounted bead (diagonal band at top), the cell membrane (orange), the top of the nucleus (blue), intranuclear punctate region (purple), and bottom of the nucleus (green). Gaussian curve fitting for each time point in the kymograph was used to track the four colored intensity lines (Supplementary Fig. 5 and Methods).

Millisecond synchronization of the AFM and camera data acquisition enabled direct comparison of displacement data from the kymograph, with force-rupture event data (Fig. 4b). The black force trace of Fig. 4b proceeds from a positive compressive force, through zero force at t ~ 1 s, to a series of adhesive force events between 1.5 and 3.4 s. The deformation data are presented as displacements relative to final position. The traces indicate that membrane displacements of several hundred nm at 2, 3, and 3.2 seconds are associated with nN changes in force (Fig. 4c, the sign of the force is flipped for ease of comparison with kymograph tracking data). As the force applied to the AFM tip increased (became more negative, Fig. 4b), the cell membrane and, to a lesser extent, the top of the nucleus were displaced upwards until the attachments to the AFM tip were ruptured, which produced a drop in force applied to the AFM tip (became more positive, Fig. 4b) and a drop in membrane height. These force rupture peaks were concurrent with membrane motion to within the 20 ms time resolution accessible at this frame rate (Fig. 4c). Moreover, the upward displacement of the cell membrane dropped in synchrony with two of the force-rupture peaks, implying that these force-rupture events were the result of membrane detachment from the AFM tip.

There are several peaks in the force-rupture pattern that do not produce features in the kymograph data. Further analysis was performed to capture deformation in locations not included in the kymograph. Regions of displacement within the cell corresponding to specific force peaks were identified with difference imaging (Methods). Figure 5 presents the retraction portion of the same force-indentation curve as Fig. 4 with side-view images taken simultaneously with the final rupture events. Areas of structural change determined from image difference analysis are shown in green. All force rupture peaks identified in Fig. 5a (B,C,D,E,F) were associated with cell membrane motion; however, only the final two peaks (E,F) resulted in nuclear motion. This was a surprising result and suggests that some of the adhesions had direct mechanical connectivity to the nucleus while other adhesions did not.

Peak E consisted of the largest total force drop of 13.2 nN (between point E and F in Fig. 5b) and the most locations with observable displacements (Fig. 5b inset), most notably motion of the nucleus boundaries and intranuclear punctate regions. Peak E rupture occurred through 5 smaller force drops identified as E0, E1, E2, E3, and E4 in Fig. 5b, with corresponding synchronized image frames indicated in Fig. 5c. For these images, difference imaging was performed between subsequent frames. At peak E0, a short-lived extension of fluorescently-labeled membrane was observed, which broke from the tip, resulted in a 0.9 nN force drop, and was no longer prominent in the following frame (E1). A more detailed view of the extension, measured at a length of 1.8 μm, is displayed in Supplementary Fig. 7. Only peaks E1 and E4 corresponded to membrane motion in the location addressed in the kymograph (Fig. 5); however, all sub-peaks exhibit cell membrane and nucleus motion. The final rupture peak, Peak F, corresponds to a final detachment of the AFM tip from the cell membrane. Peak F was associated with a sudden drop in the cell membrane and nucleus region directly under the AFM tip.

## Discussion

Our demonstration experiments in cellular indentation and adhesion show the capability of the AFM - PRISM/VLS system for providing new insights into cell mechanics. In our indentation experiments, we observed that the onset of nuclear deformation was correlated with a change in cell stiffness. These measurements are consistent with finite element models of AFM induced deformation of an elastic material with harder inclusion (i.e., the nucleus) which show the force-indentation curve deviates from a single Hertz model fit at a distance that reflects the indentation depth of the inclusion^37,38^. This is also consistent with several studies that have shown that the nucleus is stiffer than the cytoplasm^16,39^. Though these were not the first AFM measurements to report multiple stiffness regimes in cell indentation experiments^37^, these preliminary results are the first to have synchronous image data that reveal the structural origins of depth-dependent stiffness. The high speed, side-view imaging allowed for distinction between the compression of the cytoplasm associated with an initial stiffness and the subsequent compression of the nucleus contributing to a higher apparent elastic modulus. The onset of nuclear deformation during external compression has implications for a range of phenomena including force modifications in gene expression^40^ and in nuclear envelope disruption^41^. There is a need for more accurate mechanical models of cell deformation that move beyond the homogeneous elastic medium approximation and include viscoelastic contributions from multiple mechanical elements within the cell. The nucleus^16^, the cortical actin^37^, and the glycocalyx^9^ have all been proposed as playing a role in deviations from simple Hertzian force vs. indentation response. Validating the viscoelastic contributions of each element will require high resolution structural data coupled with force measurements taken with sufficient time resolution to capture dynamic response. We anticipate that methods like VLS/PRISM that provide these capabilities in side view for AFM compression measurements will greatly facilitate refinement of more detailed and accurate mechanical models.

In the adhesion experiments, we observed two categories of force rupture events: those that produced nuclear motion along with membrane motion, and those that resulted in membrane motion only. All force rupture events, ranging from 0.9 to 7 nN, resulted in measurable membrane motion of up to several microns. In similar AFM experiments employing 5 um fibronectin coated beads, focal adhesion rupture forces have been shown to be contact time dependent, with forces rising from a few nN at ~2 min to up 40–50 nN at contact times beyond 5 min^42,43^. Focal adhesion maturation time, measured by observing recruitment of FA proteins, has been shown to occur over many minutes^44^. Our contact time of ~10 s prior to pull away is relatively short and likely corresponds to the earliest stages of focal adhesion formation, and our rupture forces are correspondingly lower than those measured for mature adhesions (but much higher than for our controls on uncoated beads. See Supplementary Fig. 8). Our data suggest that even in the first few seconds after the fibronectin-integrin interaction is initiated, robust mechanical linkage between ECM and the interior of the nucleus is already in place.

Only the final rupture peaks E and F resulted in measured motion in the nucleus, which indicate that, despite the rupture of the membrane attachments during initial force-rupture events, the key attachments linking directly to the nucleus were unaffected. Moreover, the strongest interactions between the fibronectin coated tip and cell membrane formed direct linkages to the nucleus. Larger rupture events (≥2 nN) sometimes produced nuclear and intranuclear motion, and smaller rupture events (<2 nN) only resulted in membrane motion. This suggests that stronger adhesions are more likely to form direct mechanical linkages to the nucleus. There is increasing interest in the direct mechanical connection between adhesions at the cell interface with the extracellular environment and the interior of the nucleus^6,20^. Integrin mediated adhesions^45^ have been shown to couple external forces via the cytoskeletal network directly to LINC complexes at the nuclear envelope^19,46,47^ which in turn couple mechanically to nuclear lamina and chromatin. External mechanical stimuli applied via this linked mechanical network can affect nuclear mechanotransductive pathways^6,48^. Disruption of this force transmission conduit from external stimuli to nucleus has been implicated in impaired cell polarization and motility^49^ as well as disease states such as muscular dystrophy^50,51^. In this preliminary study, we demonstrated through synchronized force and sideways imaging, the capability to distinguish individual adhesions that form direct linkages to the nuclear interior from those that don’t. This opens up the potential to follow the mechanical signal from cell exterior to chromosome at the individual molecular interaction level: integrin to cytoskeleton to LINC complex to lamina to chromosome. Such experiments would provide invaluable in mapping out a fuller understanding of normal and pathological modes of nuclear mechanotransduction. More broadly, the combined AFM – VSL/PRISM system we’ve developed can provide particular insight where correlation between force and protein localization or subcellular rearrangement is critical to addressing key questions in mechanobiology. We are currently developing a second generation system that incorporates four colors and volumetric imaging.

## Methods

### Vertical Light Sheet Optics

The VLS systems was built externally to the microscope body and operated in conjunction with the microscope’s tube lens and imaging objective to produce the desired light-sheet dimensions (Supplementary Fig. 2a). The light source is a 532 nm diode-pumped solid state green laser diode (Thorlabs, Inc., DJ532-40) with a circular beam output. The light passes through a series of spherical and cylindrical lenses to produce a beam with a sheet cross section (i.e., >100 μm along the long axis and ~1 μm along the thin sheet axis). Each cylindrical lens acts to focus the beam only along one axis perpendicular to the light path. In Supplementary Fig. 2a, the beam behavior in orthogonal directions is denoted by red and blue lines. Unless otherwise noted, all lenses were purchased form Thorlabs. Component details: L_1_ (f = 4.0 mm, 352610-A), L_2_ (f = 125 mm, LA1986-A), C_D1_ (f = 25 mm, J1075L1-A) C_D2_ (f = 150 mm, LJ1629RM-A) C_D3_ (f = 100 mm, LJ1567RM-A), C_W1_ (f = 200 mm, LJ1653RM-A), C_W2_ (f = 75 mm, LJ1703RM-A), PBS (polarizing beam splitter, PBS251, Thorlabs), Specimen and Control Objective (Olympus, 1.2 NA water, UPlanSApo), Quarter wave plate (l/4) (532 nm, WPMQ05M-532), Dichroic (TRF59904, Chroma Technology Corp).

The blue lines correspond to beam along the direction of the sheet’s thin axis at the specimen, labeled “d_s_” in Supplementary Fig. 2a inset. The beam along the thin axis is affected by cylindrical lenses C_D1_, C_D2_ and C_D3_ as well as all spherical lenses (L_1_, L_2_, Tube lens and both objectives). The red lines correspond to the beam along the direction of the sheet’s wide axis (“W”). The beam along the “W” axis is affected by cylindrical lenses C_W1_ and C_W2_ along with all spherical lenses. The first two lenses, L_1_ and C_D1_, are spaced apart a distance equal to the sum of their focal lengths and act as a tube lens on the thin axis, while, L1 and CW1, act as a tube lens on the sheet width “W” axis. The lateral mirror, placed in a 4 f configuration, permits lateral movement of the light-sheet without affecting other light-sheet properties. We employed an optical technique of axial displacement presented by Botcherby *et al*.^52–54^. By moving the axial mirror, the height of the focused sheet waist is adjusted without introducing spherical aberration. The entire system – AFM, optical microscope, and light-sheet optics – fits inside a BCH-45 acoustic enclosure and a Herzan AVI series vibration isolation platform (Asylum Research).

#### Characterization of VLS illumination

The beam width was measured as a function of depth by scanning fluorescent beads through the light-sheet using the AFM scan stage and plotting max intensity as a function of position. The specimen was created by drying a sparse dilution of fluorescent beads in ethanol onto a glass coverslip. The sample was then loaded onto the AFM scan stage and was passed through the light-sheet in increments of 0.5 μm. For each image, the intensity profile of each bead was determined via fitting to a Gaussian distribution. These intensities were plotted as a function of scan stage position, and then fit to a Gaussian to determine the full width at half maximum (FWHM) as a measure of sheet width (Supplementary Fig. 2b). The axial mirror position was then moved, and the process was repeated. The result was a beam waist of 0.9 μm and a corresponding depth of focus (DOF, equal to twice the Rayleigh length) of ∼10 μm for our system.

Supplementary Fig. 2c compares the light-sheet produced by our system with that of another published light sheet system^33^ designed for single cell measurements. The theoretical FWHM of the beam for each system is depicted by a dashed line. The light-sheet produced by Gebhardt *et al*.^33^ for single-molecule imaging was controlled by the size of the aperture on the back of the illumination objective. Increasing the aperture size reduced the size of the beam waist; however, this also resulted in a decrease of the DOF. Depicted in Supplementary Fig. 2c are sheet profiles produced by two aperture diameters in the Gebhardt system (black = 12 mm and blue = 4 mm) and our system (red). The large aperture configuration produces a beam waist narrower than our VLS; however, the DOF is only a few microns and would not be useful for full cell measurements. In contrast, the smaller aperture configuration produced a beam waist slightly larger than ours and with a very large DOF. The latter configuration was used for all single-molecule transcription factor studies in the Gebhardt paper^33^ as it consistently illuminated a large enough section of the specimen.

To test the change in image quality between broad illumination and VLS illumination in PRISM images, 20 nm beads were incubated with ovarian cancer cells (SKOV3) to serve as point sources that could be analyzed in the PRISM images. The resulting plan- and side-view images can be seen in Supplementary Fig. 3. The plan-view image shows a false color overlay of broad (red) and VLS (green) illumination. The broad illumination makes it apparent that the entire cell is decorated with 20 nm beads that contribute to out-of-focus fluorescence, while the sheet illumination excites a thin band of the cell several bead-diameters thick. To produce PRISM side-view images, the objective focus was raised until the imaging plane was rotated by the 45° reflecting optic to acquire a side-view image of the cell. Raising the objective to change the imaging plane also increased the height of the VLS so that the beam waist was well above the cell. Therefore, before acquiring VLS/PRISM images, the light-sheet was returned to the specimen using the axial mirror. Adjusting both the axial and lateral positions of the VLS is crucial for achieving high quality side-view images with the PRISM imaging system.

To quantitatively characterize the change in signal, a plot profile through the same region of the cell for each illumination method was compared (lower panels of Supplementary Fig. 3). While the maximum intensity measured through beads within the cell were unchanged, the nucleus region exhibited a drop in out-of-focus intensity by over 50%.

### PRISM-AFM integration

The PRISM reflecting optic must fit within the vertical space between the AFM cantilever holder and the coverslip when the AFM tip is fully engaged, shown in Supplementary Fig. 1. The unmodified clearance is approximately 300 μm, but ultimately depends on the choice of cantilever and the placement of the AFM cantilever chip in the holder. To increase this distance, shims were added below the cantilever chip and spring clip (see Supplementary Fig. 1). Moving the cantilever further from the window resulted in less than a <10% reduction in the AFM quadrant photodiode signal for up to 500 μm shim thickness. The cantilever holder, modified with shims below the cantilever chip and spring clip, allow for a clearance of nearly 1 mm.

The 45° reflecting optic and mount were chosen so that they would fit in the allowed space and have clearance to move vertically. A 180 μm right-angle prism with coated hypotenuse (Precision Optics Corporation, MA) was adhered to a rectangular cross section capillary tube (Wale Apparatus SKU:5010-100) with UV-cured Norland Optical Adhesive 81 (Norland Products Inc., NJ). The total height of the capillary tube (300 μm) and micro-prism (180 μm) is approximately 500 μm with adhesive, which easily fits in the nearly 1 mm clearance allowed between cantilever holder and glass coverslip, as illustrated in Supplementary Fig. 1. The prism - capillary tube assembly is attached to a 6 DOF translation stage for ease of positioning of the PRISM near target specimens. The front portion of the capillary tube was shaved back prior to prism attachment to avoid interference with the light path of the AFM’s cantilever tracking super luminescent diode (SLD). (Supplementary Fig. 1). As described below, long AFM cantilevers (>450 um) were required to provide an unobstructed path for the reflected SLD light.

### Imaging Plane Alignment with VLS

The imaging plane and light sheet plane need to be parallel for optimum imaging quality. The 6 DOF translation stage enables adjustment of the PRISM-capillary cantilever assembly orientation and in turn the effective angle of the PRISM for imaging. For an individual PRISM mounting, some adjustment to the angle of the capillary cantilever is made to optimize PRISM imaging prior to performing an experiment. This optimization is accomplished by adjusting the PRISM angle until the relative tilt of the light sheet and focal plane is minimized, apparent from uniformity in the illumination of the sample as the focal plane is swept through the light sheet plane.

### Synchronization of Image and Force Data

Synchronization of high-speed imaging and AFM data acquisition was accomplished by utilizing the AFM controller as an external trigger for the Orca Flash 4.0 camera. A voltage pulse wave command was inserted into the AFM’s standard constant-velocity force curve IGOR control code, and was sent via the AFM controller BNC output to the camera’s external trigger input. The force and image data were synchronized to better than 1 ms. This resolution was determined by pulsing the SLD which provided a precise and clear signal in both the force (via the quadrant photodiode) and image data (See Supplementary Fig. 9).

### PRISM-Image Resolution

To measure the resolution of the side-view images acquired with PRISM, the point spread function (PSF) of sub-pixel microspheres was measured. Characterization samples were prepared by incubating 20 nm red fluorescent beads with SKOV3 cells, then rinsing and fixing the sample using formaldehyde. Two PSF stacks were acquired for each bead analyzed on the system – a plan-view z-stack and a side-view x-stack. Then, each image stack was analyzed by fitting a Gaussian function to the intensity profile in each axis direction through the maximum intensity pixel, and then determining the full width half maximum (FWHM).The results for plan-view and PRISM-view PSF characterization of a single bead are shown in Table 1. These values were within 15% of the theoretical FWHM for the system. Similar results were achieved for 10 other beads for which plan-view and PRISM-view stacks were acquired (See Supplementary Table 1).

**Table 1:** Point Spread Function for Vertical Light Sheet.

PSF	Plan View FWHM	PRISM-View FWHM
X	332 nm	870 nm
Y	305 nm	357 nm
Z	947 nm	321 nm

### AFM and Bead Attachment

All force data was collected with an Asylum MFP-3D Bio AFM (Asylum Research, Santa Barbara, CA). 5 µm polystyrene beads (Corpuscular, NY) were attached with Norland Optical Adhesive (Norland, NJ) to Arrow-TL1 tipless cantilevers (nominal k = 0.03 N/m, Nanoworld). Cantilever spring constants were calibrated using the thermal tuning method. The AFM cantilever was loaded into the AFM cantilever holder and 10 µL of 0.01 mg/mL fibronectin in PBS was placed on the end of the cantilever where the bead was attached. The solution was allowed at least 15 minutes to physisorb onto the polystyrene bead before rinsing with medium and loading into the AFM head.

### Sideways imaging and AFM force collection

Single SKOV3 cells were selected that were firmly attached to the gel, exhibited spreading behavior representative of the cell line, and were sufficiently fluorescent in plan-view. Next, the AFM bead tip was positioned over the nucleus of the selected cell. The AFM bead and cell were then jointly positioned such that the VLS illuminated the region of the cell directly under the AFM bead. Finally, PRISM was positioned ∼50 µm from the AFM bead to avoid contacting the cell or AFM cantilever. The imaging objective height was then raised to find the pathway rotated side-view imaging plane (Supplementary Video 2). Adjustment of the height of the objective also changes the vertical position of the narrowest part of the light sheet, such that VLS and cell are no longer aligned. The VLS was translated back down to the cell using the axial adjustment mirror in the VLS optics (Supplementary Fig. 1). Once the imaging and illumination planes were aligned, the IGOR software was used to acquire a standard constant-velocity force curve and externally trigger the ORCA Flash4.0 CMOS camera (Hamamatsu Photonics K.K., Japan). Images were taken at 50 fps.

### AFM Data Analysis

AFM data were typically collected at 100 kHz. Force curves were analyzed with custom MATLAB code to calculate the Young’s modulus using the Hertz model. Briefly, the program identifies the contact point coordinates using a golden-section search, which attains the minimum total fitting error for a linear fit of the data prior to the contact point, and a Hertz model fit to the data from the contact point, up to a user defined maximum indent. Once the contact point is found, least-squares fitting is applied to the force-indention data in the post-contact region of the force curve to the Hertz model to extract an effective Young’s modulus. For fitting two regimes of stiffness, a “two Hertz” fit was performed in which the Hertz fitting was applied separately at low indentation and at high with an additional parameter, the point of transition between regions (I and II), optimized to minimize error in the two fits.

### Gel Preparation

All experiments were done on polyacrylamide gel surfaces. This was done to avoid imaging artifacts, including the reflection of fluorescent label off the glass coverslip in side-view images and imaging difficulties associated with imperfections or reflective-coating issues on the bottom edge of the micro prism. All reagents were purchased from Thermo Fisher Scientific (Liverpool, NY) unless noted otherwise. Gels were prepared to achieve 50–60 kPa stiffness, using a ratio of the 4:3:3 50 mM HEPES, 40% acrylamide, and 2% methyl-bis. Gelation was activated by the addition of N,N,N′,N′-tetramethylethylenediamine-1, 2-diamine, and 10% ammonium persulfate. The gels were prepared onto 24 × 50 mm glass coverslips (Corning #1.5), previously UV-cleaned and then vapor treated with 1,3,5,7-tetravinyl-1,3,5,7-tetramethylcyclotetrasiloxane. After catalyst addition, 10 µL of gel solution was placed in the center of the coverslip and rapidly covered with a HMDS (hexamethyldisilazane) vapor-treated 22 × 22 mm coverslip to compress the gel and spread it evenly on the coverslip. The gel was allowed to partially dry in a sterile biological safety cabinet before removing the 22 × 22 mm coverslip from the top of the gel.

To attach fibronectin to the gel, sulfo-SANPAH (N-sulfosuccinimidyl 6-(4′-azido-2′-nitrophenylamino) hexanoate) was dissolved in DMSO (dimethylsulfoxide, cell culture grade) at ∼1 mg/mL and spread into a 1 cm diameter circle with a pipet tip in the center of the gel. The excess solution was removed from the gel sample. Then with the gel inside the biological safety cabinet, the UV light was turned on for ∼10 minutes or until the sulfo-SANPAH color turned from orange-red to slightly brown. The sulfo-SANPAH was then rinsed with 3 successive applications and removals of PBS (phosphate buffered saline). Then 0.01 mg/mL fibronectin in PBS was placed onto the same location with enough solution to barely cover the same area. The UV light was again turned on for 5 minutes to allow crosslinking of the fibronectin onto the gel surface. After removal of the fibronectin solution and while relatively dry, a 1 cm glass cloning cylinder was added to the gel with thin layer of vacuum grease and sterile cell media was used to rehydrate the gel. The vacuum grease serves the dual purpose of preventing buffer leakage during overnight incubation and AFM-PRISM experiments.

### Cell Culture

Cells were plated onto the gels by trypsinization of a 50–90% confluent culture of SKOV3 human ovarian cancer cell lines (from Gerard Blobe’s lab at Duke University). The cultures were diluted back to about 30% confluence, and then an aliquot of cells was further diluted by at least 1/10–1/30 before plating in RPMI (Roswell Park Memorial Institute) medium with 5% serum and antibiotic/antimycotic at 1x. Cells usually attached within several hours, but were incubated overnight to ensure firm attachment.

On the day of the experiment, cells were stained with vital (live-cell) stains for SYTO 83 (Molecular Probes) nucleic acid label, and Vybrant (ThermoFisher, CM-Dil, V22888) membrane label. The sample was rinsed profusely with RPMI medium by removing liquid from the cloning ring, replacing with new medium, and repeating several times to remove loosely attached cells. A larger secondary ring of vacuum grease was added onto the gel surface concentric to the cloning ring. The majority of solution was removed from the cloning ring, and the cloning ring was removed from the gel surface. Finally, the sample was loaded onto the AFM stage and additional medium was added to form a small bubble in the inner vacuum grease ring.

### Image Analysis

Motion of the cell membrane and subcellular components were measured using kymograph and difference image analysis.

#### Kymograph Analysis

A vertical ROI directly below the AFM tip was selected (Supplementary Fig. 5a). Kymographs of image stacks were created with the ImageJ KymographyReslice plugin (Supplementary Fig. 5b). Using custom MATLAB code, the positions of the intensity peaks of the kymograph were tracked using Gaussian fitting (Supplementary Fig. 5c), with initial estimates of peak positions provided manually by the user in the first frame. The result of tracking the bright regions with the Gaussian tracker can be seen in Supplementary Fig. 5c.

#### Difference Imaging

Image differencing is an image processing technique used to identify changes between images, calculated by finding the difference between each pixel in two images, and generating a resulting difference image. To identify regions of the cell with motion correlated to force-rupture events (Fig. 5), difference images were taken between an image acquired just prior to the force-rupture peak and an image after the peak. An interval of at least 3 frames (60 ms) between the image frames was used in the difference analysis. The images were subtracted, and a threshold was then applied to reduce the background signal. This threshold level was chosen by assessing the background noise level and the difference signal coming from regions of noise (far from cell movement). The threshold was set such that the majority of the difference signal coming from this background noise was eliminated. Finally, an area filter was imposed to further ensure that only those regions that showed significant intensity changes were selected. The area filter averages a pixel with its neighboring pixels. This filter effectively removes signal from the difference map where large difference signal occurs over very small areas and is likely due to large random fluctuations and unlikely due to real cell deformation. The difference image data is highlighted in green in Fig. 5b & c.

### Data availability

The datasets generated during and/or analyzed during the current study are available from the corresponding author on reasonable request.

## Electronic supplementary material

Supplementary Material

Supplementary Video 1

Supplementary Video 2

Supplementary Video 3

Supplementary Video 4

Supplementary Video 5
