# Supplementary material for: Vertical Light Sheet Enhanced Side-View Imaging for AFM Cell Mechanics Studies
Source: Sci Rep. 2018 Jan 24;8:1504. doi: 10.1038/s41598-018-19791-3 (PMC5784156; doi:10.1038/s41598-018-19791-3)
Supplement: Supplementary file 1 — Supplementary Material [file 41598_2018_19791_MOESM1_ESM.pdf]

Vertical Light Sheet Enhanced Side-View Imaging for AFM Cell Mechanics Studies

Supplementary Information

Kellie Beicker[1], E. Timothy O'Brien III[1], Michael R. Falvo[1], Richard Superfine[1]*

[1]Department of Physics and Astronomy, UNC-Chapel Hill, Chapel Hill, NC

*Correspondence to rsuper@physics.unc.edu

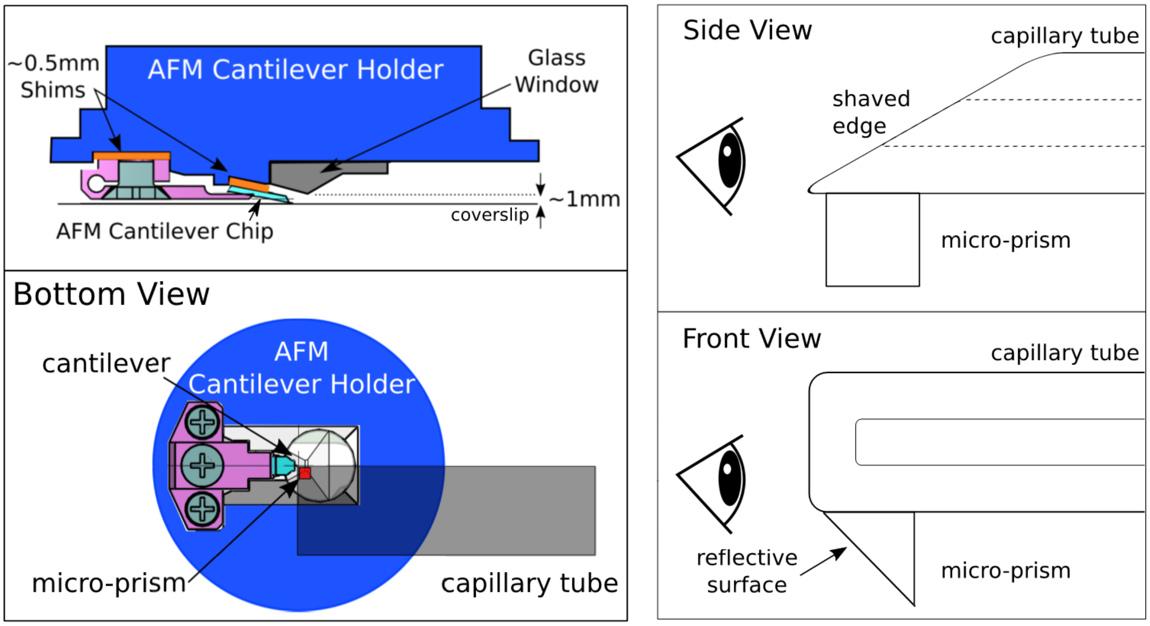

Supplementary Figure 1. Modifications to AFM cantilever holder and micro-prism mounting. (Top-Left) Side-view cutaway image of cantilever holder with ~1 mm clearance between the glass window (grey) and glass coverslip due to use of shims (orange). (Bottom-Left) The bottom view of micro-prism (red) placed near AFM cantilever, as would be seen by an inverted optical microscope. All elements are drawn to scale except micro-prism, which is enlarged to clearly identify its location. (Top-Right) Side-view image of micro-prism mounted on bottom of shaved capillary tube. The eye indicates the direction from which the front-view image is drawn. (Bottom-Right) Front-view of micro-prism on capillary tube, where the eye indicates the direction from which the side-view image is drawn.

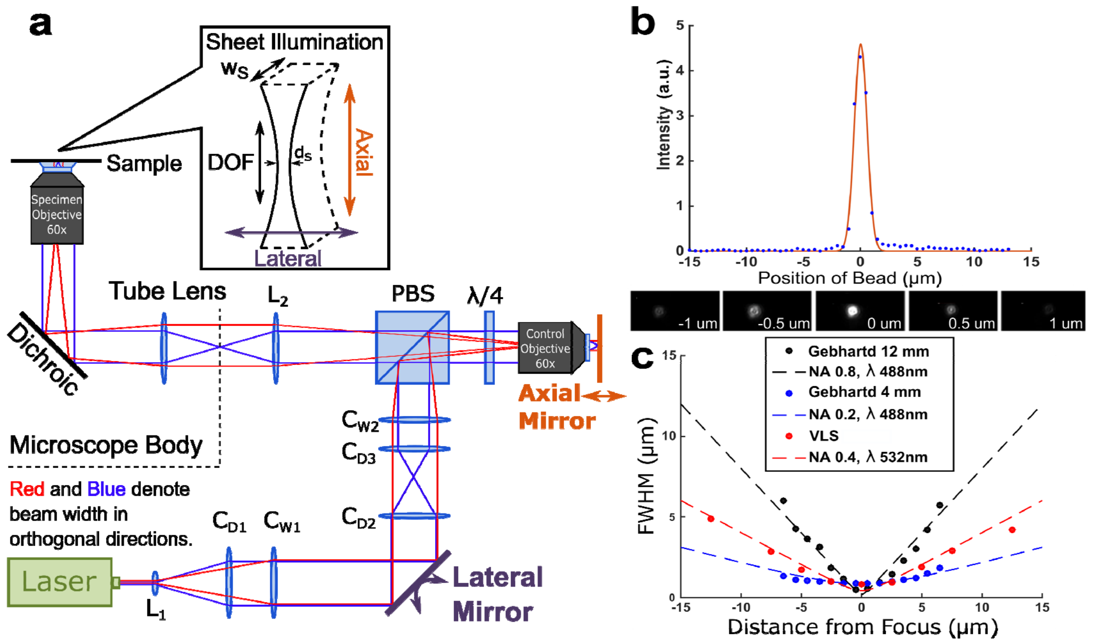

Supplementary Figure 2: Vertical light sheet designed and characterized to produce beam waist of ~1 μm. (a) The system consists of a series of spherical lenses (L_1, L_2) and cylindrical lenses oriented either along the sheet waist axis (C_{D1}, C_{D2}, and C_{D3}) or along the sheet length axis (C_{W1} and C_{W2}), control and specimen objectives, axial and lateral mirrors, and polarized beam splitter. A dichroic was used in the place of a standard plane mirror to allow excitation wavelength to be reflected and the epifluorescence emission wavelength to pass through. The lateral mirror provides lateral displacement of the laser illumination in the sample plane. The control objective and the axial mirror are used for remote axial translation of the light-sheet up and down relative to the sample plane. (b) Intensity of 20 nm fluorescent bead sample illuminated by VLS as a function of the lateral position translated by AFM scan stage. Sample images of a 20 nm fluorescent bead sample illuminated by VLS as it is translated through the sheet are shown in panels below. (c) FWHM of VLS as a function of axial mirror position, which provides a measure of sheet depth of field (see Online Methods for details)

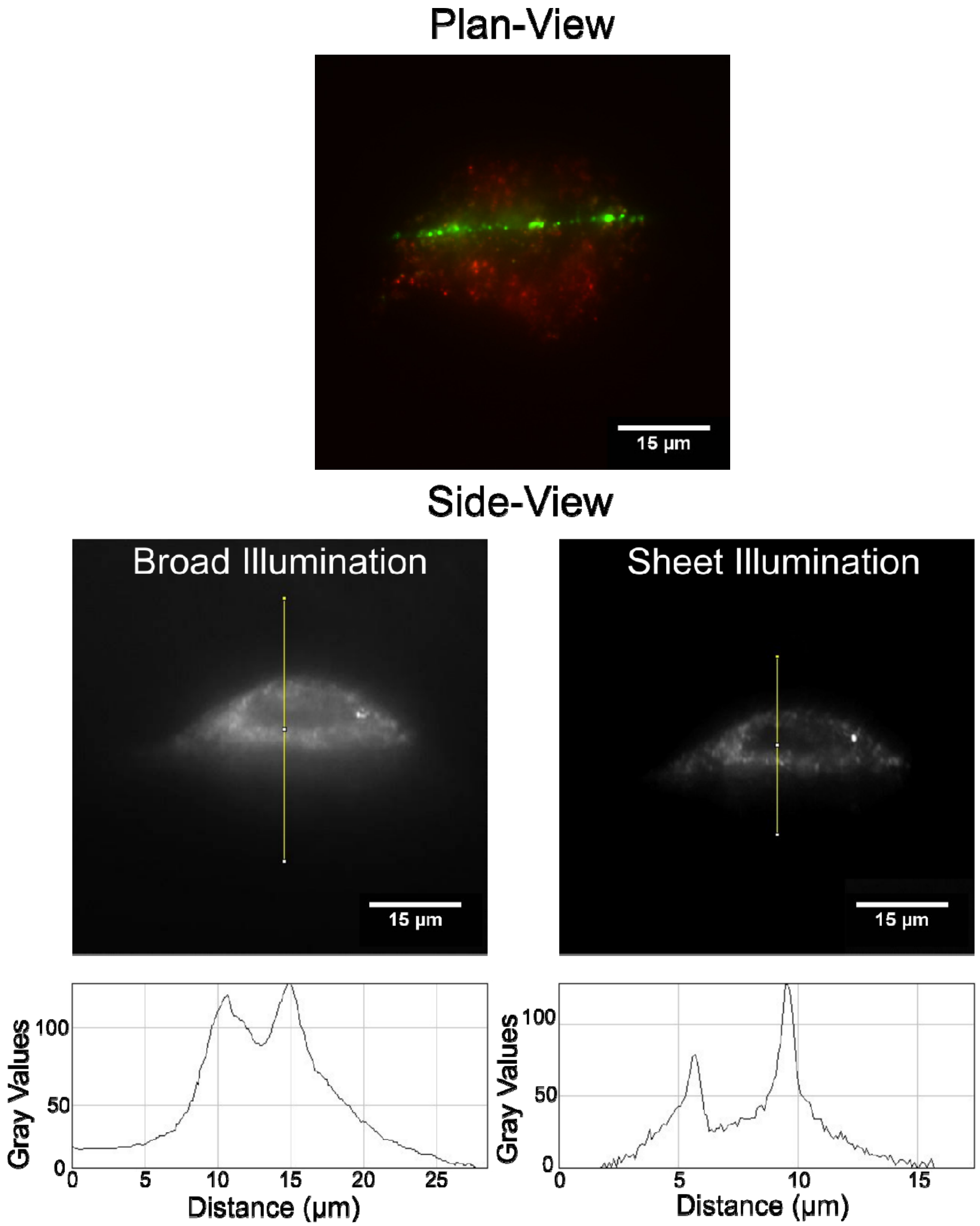

Supplementary Figure 3: Vertical light-sheet (VLS) illumination improves imaging of SKOV3 cells labeled with 20 nm fluorescent beads. (Top) False-colored plan-view image of broad (red) and VLS (green) illumination of 20 nm bead-labeled cells. (Bottom) PRISM-view images of the same cell with both broad- and sheet- illumination with the intensity profiles for the yellow line in each image.

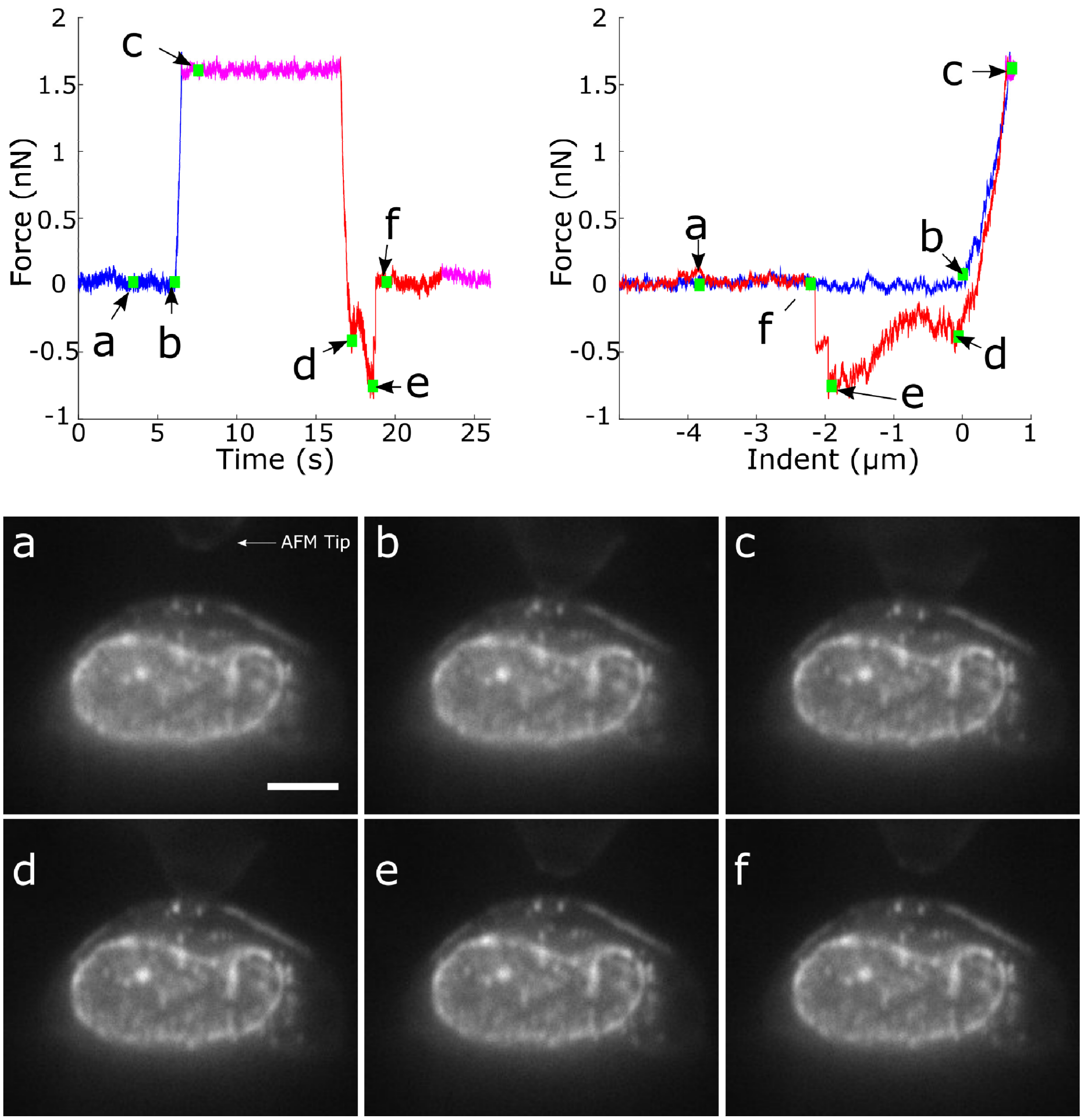

Supplementary Figure 4: Simultaneous AFM force data and images. AFM deflection and z-piezo data was converted into force and indentation to produce force-time (Top-Left) and force-indentation (Top-Right) curves, which can be partitioned into several regions - approach and indent (blue), retract and adhesion (red), and dwells at constant deflection either at the surface or away from the cell (magenta). [a-f] Simultaneously acquired images of Syto83 and Vybrant labeled SKOV3 cell at a rate of 50 fps. The locations of the images are marked in the force curves. The fibronectin-coated AFM tip was aligned over a fluorescently labeled cell and the objective was focused up to the PRISM imaging plane. Then a force curve was initialized and

simultaneous force and image data was acquired. First the AFM tip approached (a), contacted the cell membrane (b) and indented the cell to a predefined trigger force (c), where the deflection of the AFM cantilever was maintained for several seconds (magenta) before the AFM tip was retracted from the cell surface (red). Adhesion between the AFM tip and cell membrane is indicated by negative force peaks. The final rupture of adhesions between the tip and cell allowed the cantilever to return to its equilibrium position of zero force (f), which was maintained for the remainder of the retract portion of the force curve. Scale bar = 5 μm.

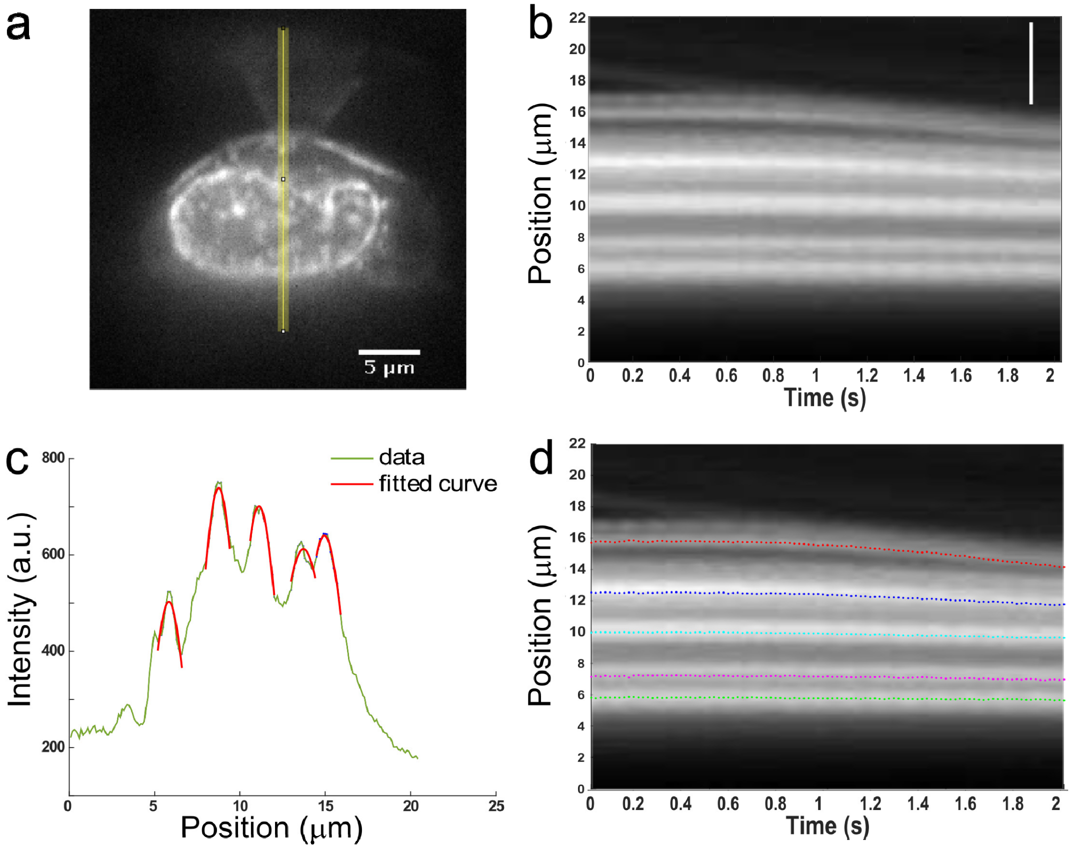

Supplementary Figure 5: Kymograph analysis of SKOV3 cell deformation images of. (a) Representative side-view image sequence with yellow vertical sliced section as the region-of-interest directly through the AFM tip center. Labeled regions of the cell - Syto 83 (nucleus) and Vybrant (cell membrane) - and the AFM bead-tip can be seen in this image. (b) Kymograph of the yellow ROI over the course of the approach portion of the dataset. Vertical scale bar = 5 um. (c) Gaussian fits to each of the 5 user-identified fluorescence intensity peaks for a single time point. (d) The result of Gaussian fits to each of the time points is overlaid on the kymograph for the top cell membrane (red), top of the nucleus (blue), and bottom of the nucleus (green). There are two additional lines (cyan and magenta) corresponding to punctate labels within the nucleus.

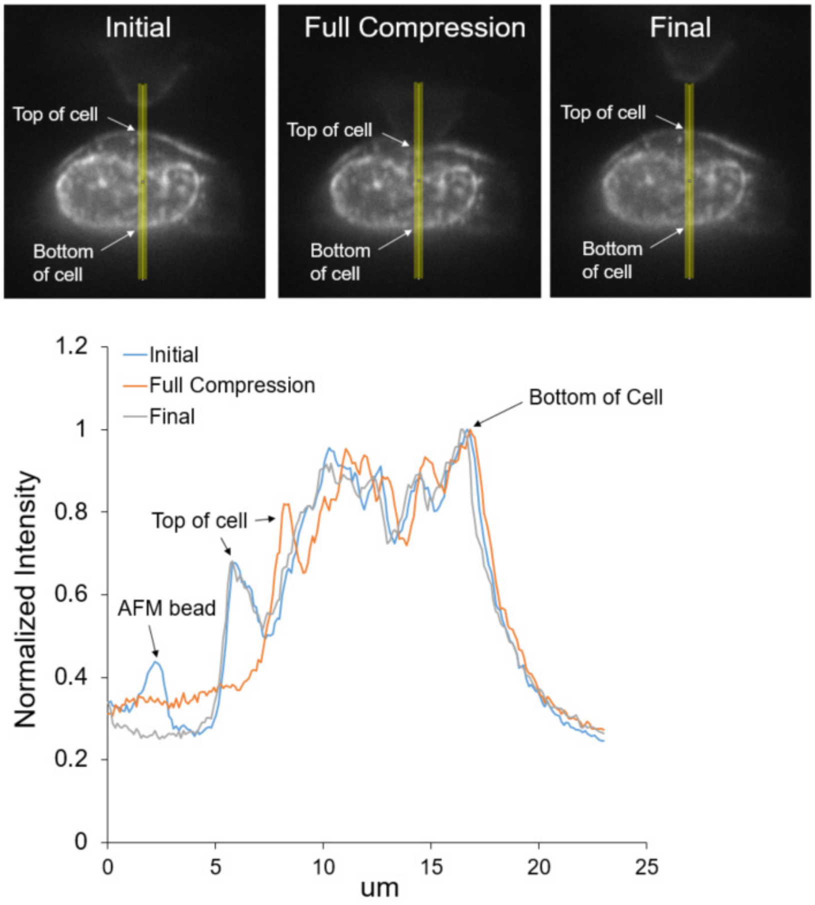

Supplementary Figure 6: Reversibility of Compressive Strain. The top three panels depict a SKOV3 cell (same experiment described in Fig. 3) before, during and after AFM bead induced compression. The yellow strip indicates the region of interest depicted in data plot below. The normalized image intensity is plotted as a function of position from top to bottom along slice depicted by the yellow strip (image data averaged across 10 pixel wide strip). Intensity data is normalized to account for a gradual decrease in intensity due to bleaching. Note that the AFM-bead is apparent in the intensity data for “initial” state (blue) as it hovers above cell. The position of the top of the cell before and after compression is very closely matched, as are all other regions of the cell. This indicates no appreciable change in cell height that would suggest plastic damage. Note also that the intensity of cell top in the fully compressed state is higher than in the before or after state, most likely due to the addition of the fluorescence intensity of the bottom of the AFM-bead at that same position.

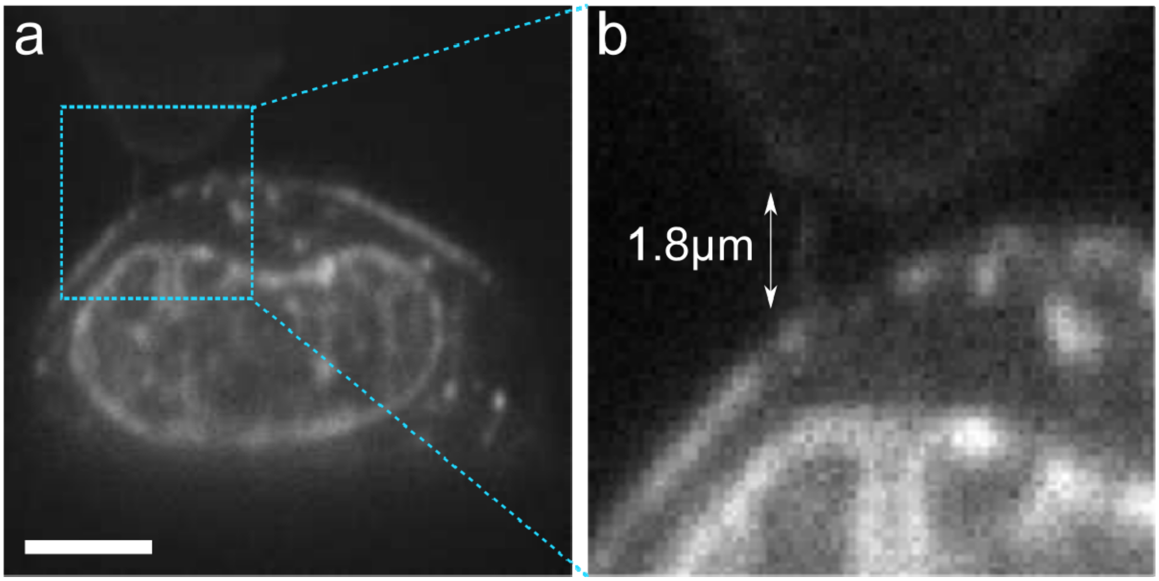

Supplementary Figure 7. Membrane extension in adhesive portion of force curve depicted in Fig. 5 of the main text. Scale bar = 5 um.

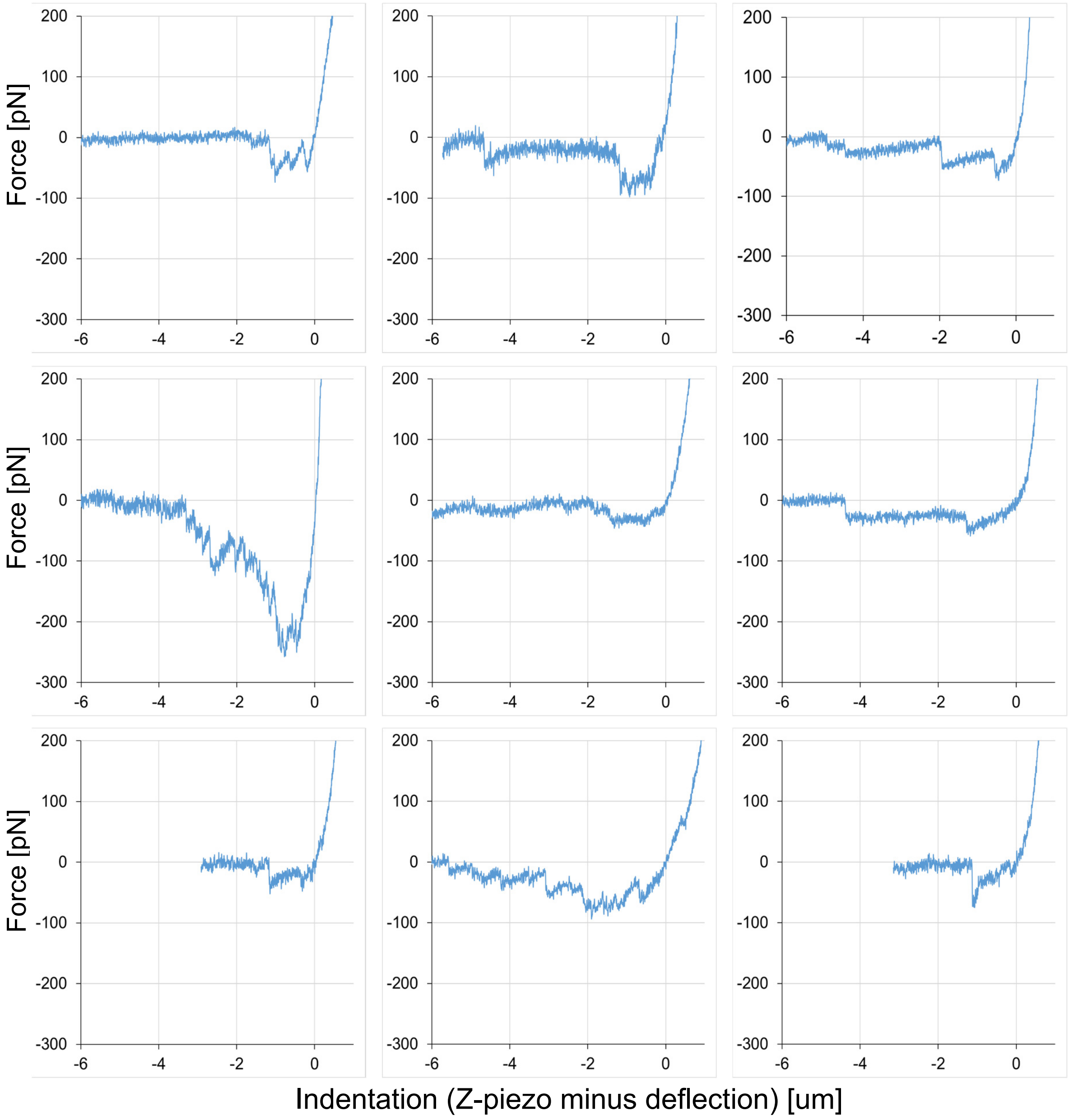

Supplementary Figure 8: Adhesion data for uncoated polystyrene beads on SKOV3 cells. The data depict adhesive forces as a function of indentation. Negative indentation here means adhesive tensile deformation, and negative forces represent the adhesive interaction between the bead and SKOV3 cell as it pulls away from the sample. Each curve represents and experiment done on a different cell. These curves are a representative sample of over 50 curves taken on the ovarian cancer cells. Note in all cases the maximum adhesive force is under 300 pN and for all but one is under 100 pN. Our observed range of adhesion forces for the fibronectin coated beads was 0.9 - 7 nN.

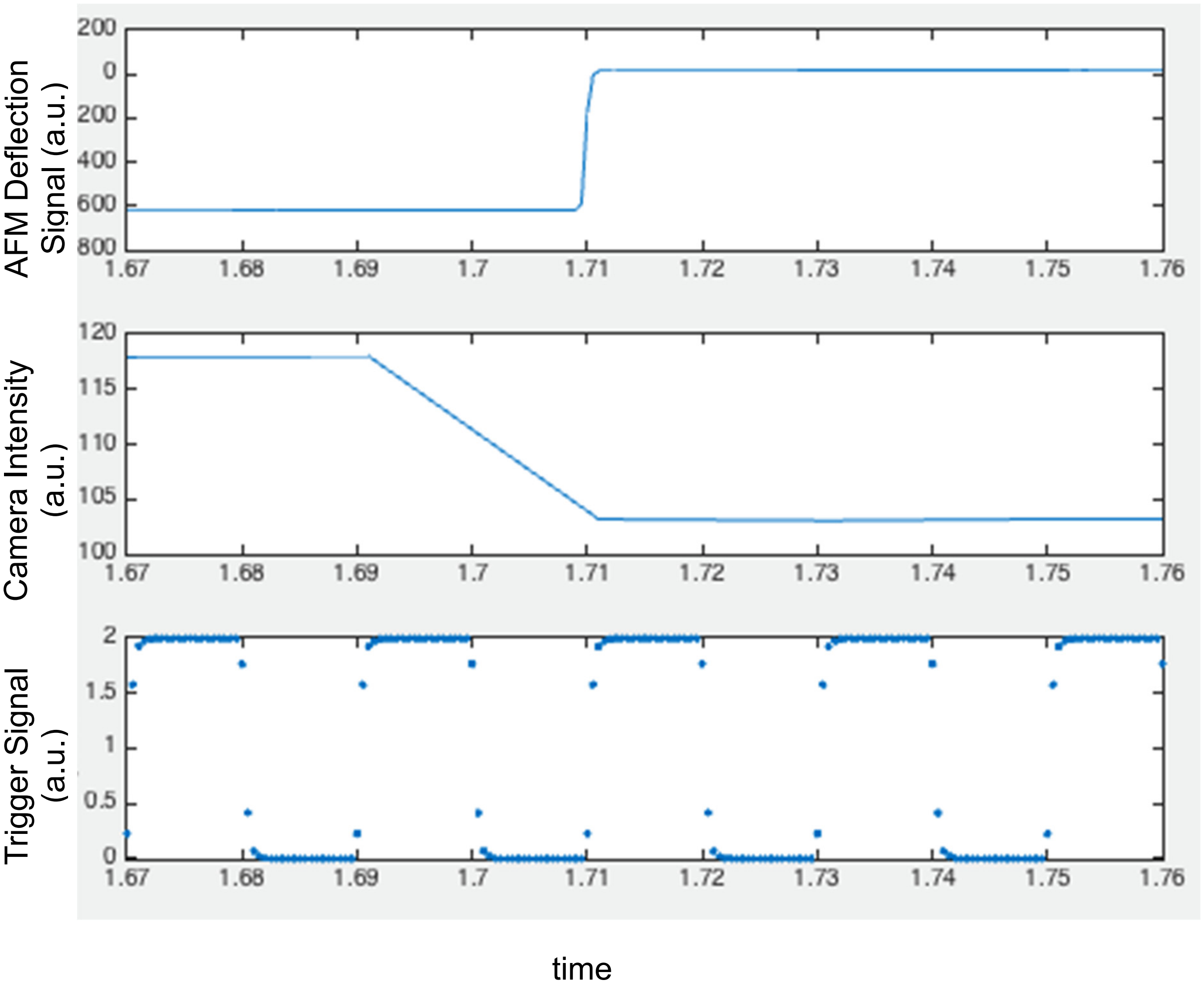

Supplementary Figure 9: Synchronization of force and camera signal. The top curve is the cantilever deflection signal (force data) coming from the AFM's quadrant photodiode. The middle curve is the intensity signal coming from the camera. The bottom curve is the pulsed trigger signal produced by AFM code. The pulse signal is produced in synchrony with force data collection and is used as an external trigger to the Orca Flash 4.0 camera. Because the camera signals on the increasing edge of the voltage signal, the force and image data was resolved to better to ~ 1 ms of simultaneous force data. This resolution was determined by pulsing the SLD which produces a signal both in the AFM deflection channel and in the camera signal. Initially the SLD is on, then turned off just before t = 1.71 s, which produces a change in the camera signal and AFM signal at the data collection occurring at 1.71 s. In practice, in the context of cell mechanics experiments, the frame rate for PRISM images of fluorescently labeled cell images was limited to 20 ms by the exposure time necessary for specific labels.

Supplementary Table 1. Resolution of PRISM sideways imaging using VLS illumination. Measurements were taken as described in the methods section. FWHM = full width half maximum of bead image intensity along indicated axis.

	**Plan – View**			**PRISM – View**		
**Bead #**	**X–FWHM [um]**	**Y–FWHM [um]**	**Z–FWHM [um]**	**X–FWHM [um]**	**Y–FWHM [um]**	**Z–FWHM [um]**
1	0.332	0.305	0.947	0.87	0.357	0.321
2	0.337	0.316	0.964	0.888	0.321	0.298
3	0.353	0.338	0.858	0.927	0.432	0.389
4	0.296	0.298	0.982	0.98	0.319	0.378
5	0.329	0.31	0.855	0.983	0.245	0.287
6	0.386	0.386	0.993	0.895	0.379	0.386
7	0.314	0.294	0.997	0.903	0.385	0.264
8	0.339	0.316	1.004	0.86	0.368	0.325
9	0.341	0.258	0.908	0.973	0.387	0.315
10	0.321	0.301	0.965	0.985	0.397	0.356
11				0.856	0.351	0.278
**Average**	**0.335**	**0.312**	**0.947**	**0.920**	**0.358**	**0.327**

Captions for Supplementary Videos:

Supplementary Video 1:

Scale cartoon movie depicting AFM VLS/PRISM system. PRISM optic is mounted to the large rectangular capillary cantilever. AFM cantilever with bead (depicted as green sphere) is tucked under PRISM optic. Green VLS beam is focused at sample plane and diverges. Adherent cells on substrate are depicted with red nuclei. The relative scale of VLS width, cells, AFM cantilever, bead, PRISM and rectangular capillary cantilever are accurate. Beam width ~ 1 micron, Bead ~5 micron.

Supplementary Video 2:

Vertical image stack showing both plan view image and PRISM side view image within the same field of view. The video starts with the plane of focus below the cell. The imaging plane is moved upward by translation of the objective. The focal plane moves up through the cell in plan view and then eventually the reflected horizontal plane of focus moves through the cell to provide the side view image. The red dotted line is a guide to the eye to indicate the approximate position of the surface in the side view image. The PRISM is oriented with its reflecting surface oriented toward the top of the image, with its base touching the substrate toward the bottom of the frame (not visible). The SKOV3 cell was labeled with membrane (Vybrant) and nuclear (Syto83) stains.

Supplementary Video 3:

Real time VLS/PRISM video of AFM indentation and adhesion measurement. A fibronectin coated bead is brought into contact with a SKOV3 cell. The SKOV3 cell is labeled with Vybrant (membrane) and Syto83 (nucleus). The AFM-bead compresses cell, dwells for a few seconds and then retracts. Note adhesive ruptures as bead releases contact with cell. Video rate is real time.

Supplementary Video 4:

Compression Measurement with synchronized AFM force and VLS/PRISM imaging data. Video in upper panel depicts a fibronectin coated bead compresses a SKOV3 cell. Lower left panel shows the indentation data during compression. The moving black dot indicates the current

position within the force data corresponding to the movie above. The lower right panel shows the adhesion data during retraction (no corresponding video). The SKOV3 cell is labeled with Vybrant (membrane) and Syto83 (nucleus). The width of the image window is 20 um. Note that real time elapsed is indicated on x axis of the Force vs. time plot.

Supplementary Video 5:

Adhesion Measurement with synchronized AFM force and VLS/PRISM imaging data. Video in upper panel depicts retraction of a fibronectin coated bead from a SKOV3 cell. Lower right panel shows the adhesion event data during retraction. The moving black dot indicates the current position within the force data corresponding to the movie above. The lower left panel shows the indentation data that occurred prior to the pull-off depicted in the video (no corresponding video). The SKOV3 cell is labeled with Vybrant (membrane) and Syto83 (nucleus). The width of the image window is 60 um. Note that real time elapsed is indicated on x axis of the Force vs. time plot.
